# Supplementary material for: A Microfluidic Platform to Monitor Real-Time Effects of Extracellular Vesicle Exchange between Co-Cultured Cells across Selectively Permeable Barriers
Source: Int J Mol Sci. 2022 Mar 24;23(7):3534. doi: 10.3390/ijms23073534 (PMC8998828; doi:10.3390/ijms23073534)
Supplement: Supplementary file 1 [file ijms-23-03534-s001.zip › ijms-1634898-supplementary.pdf]

International Journal of *Molecular Sciences*

MDPI

Supplementary Information

A Microfluidic Platform to Monitor Real-Time Effects of Extracellular Vesicle Exchange between Co-Cultured Cells across Selectively Permeable Barriers

Hunter G. Mason [1,2,†], Joshua Bush [3,†], Nitin Agrawal [3,‡], Ramin M. Hakami [1,2,*] and Remi Veneziano [3,*]

1 School of System Biology, George Mason University, Manassas, VA 20110, USA; hmason4@gmu.edu
2 Center for Infectious Disease Research, George Mason University, Manassas, VA 20110, USA
3 Department of Bioengineering, College of Engineering and Computing, George Mason University, Manassas, VA 20110, USA; jbush20@gmu.edu (J.B.); nagrawal2@cnmc.org (N.A.)
* Correspondence: rhakami@gmu.edu (R.M.H.); rvenezia@gmu.edu (R.V.)
† These authors contributed equally to this work.
‡ Current address: Center for Cancer and Immunology Research, Children's National Research Institute, Washington, DC 20010, USA.

Table S1. Measured size of liposome preparations. The average diameters of 70 nm and 250 nm Cy5-liposome preparations were measured using DLS. The diameter and polydispersity (PDI) measurements for 3 independent readings and the mean values for each preparation are shown.

Sample	Diameter nm	PDI
70 nm Liposomes 1	72.49	0.027
70 nm Liposomes 2	72.10	0.046
70 nm Liposomes 3	70.55	0.046
70 nm Liposomes Mean	71.71	0.040
250 nm Liposomes 1	249.90	0.281
250 nm Liposomes 2	258.20	0.294
250 nm Liposomes 3	246.10	0.307
250 nm Liposomes Mean	251.40	0.294

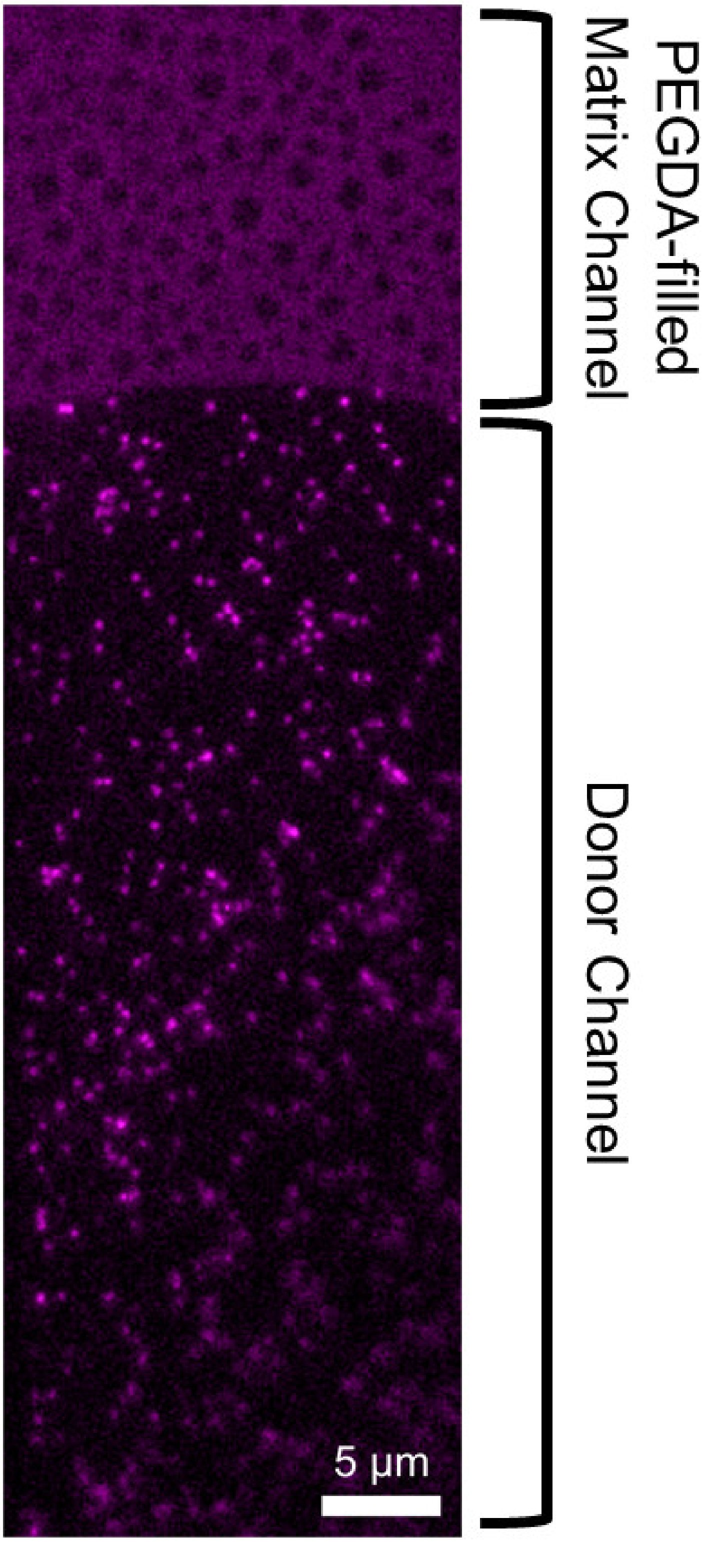

Figure S1. PEGDA hydrogel interface prevents diffusion of exosomes. The inability of exosomes to pass through PEGDA at 1.2 nm in diameter was verified using PEGDA-loaded chips that were injected with DOPE-PEG(2000)-N-Cy5 labelled U937 exosomes and visualized after 24 hours at the interface between the exosome-injected donor channel (Bottom) and the PEGDA filled matrix channel using Cy5 emission filter (original magnification: 1000×).

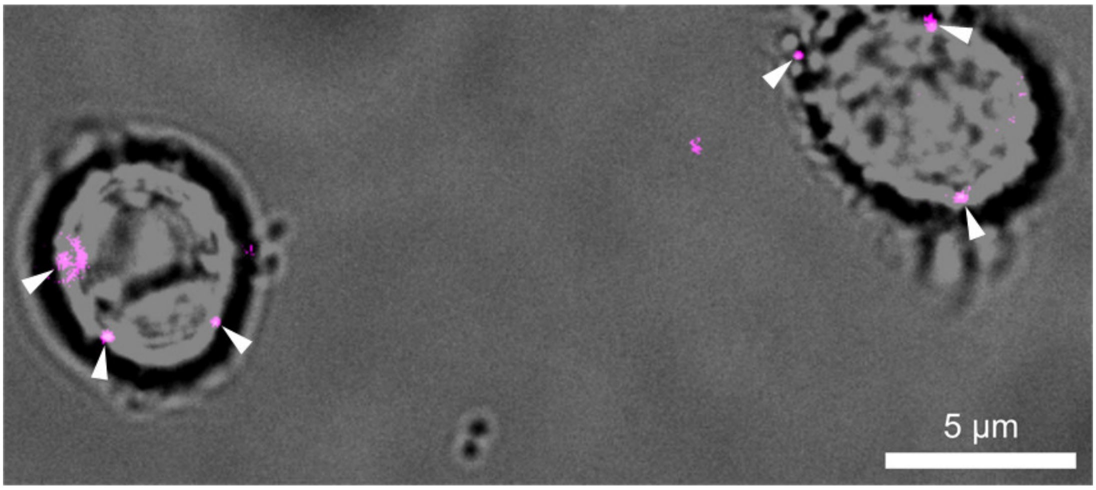

Figure S2. Verification of exosome uptake into recipient cells at 12 hours. The exosome uptake by cells in our Matrigel chip assays was confirmed by demonstrating diffusion of labelled exosomes across the Matrigel and subsequent uptake into recipient cells. A Matrigel chip was injected with DOPE-PEG (2000)-N-Cy5 labelled U937 exosomes in the donor channel and recipient U937 cells in the recipient channel. Chips were visualized at 12 hours post injection using Brightfield and Cy5 emission filters, demonstrating uptake of exosomes by the recipient cells (arrowheads point at imaged exosomes that are either inside the cells or are interacting with the cells on the surface. Original magnification: 1000×).

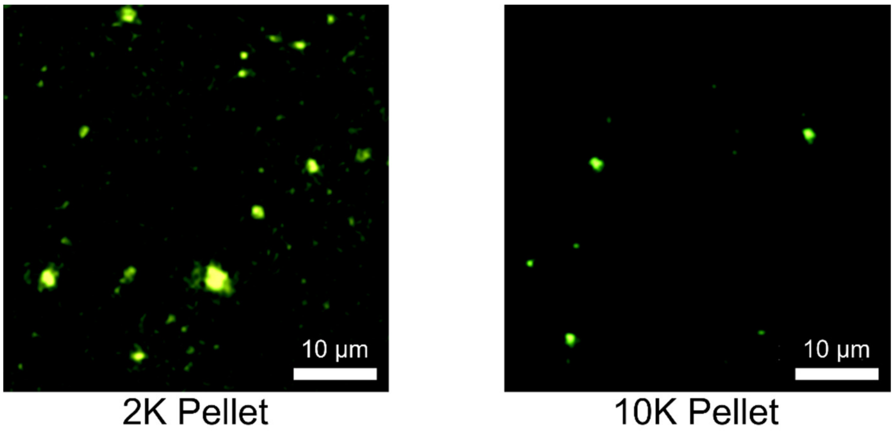

Figure S3. Visualization of GFP-tagged vesicle populations in 2K and 10K pellets. U937-XP cell conditioned media was centrifuged at 2,000 xg and 10,000 xg to collect the 2K and 10K pellets respectively. The collected pellets, which contain a greater proportion of vesicles larger than exosomes, were imaged on glass slides using a GFP emission filter (original magnification: 1000×.).

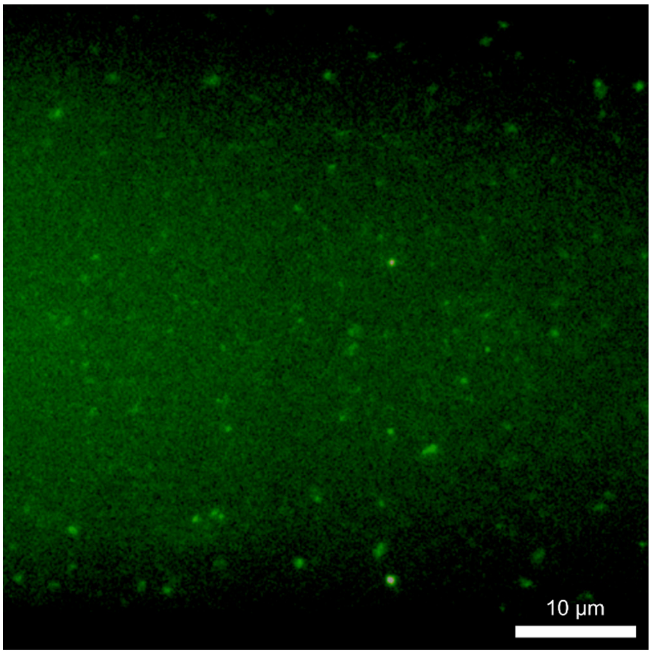

Figure S4. Accumulation of U937-XP extracellular vesicles within the Matrigel. A Matrigel chip was injected with U937-XP cells in the donor channel and the EVs in Matrigel were visualized at 48 hours with a GFP emission filter (original magnification: 1000×).

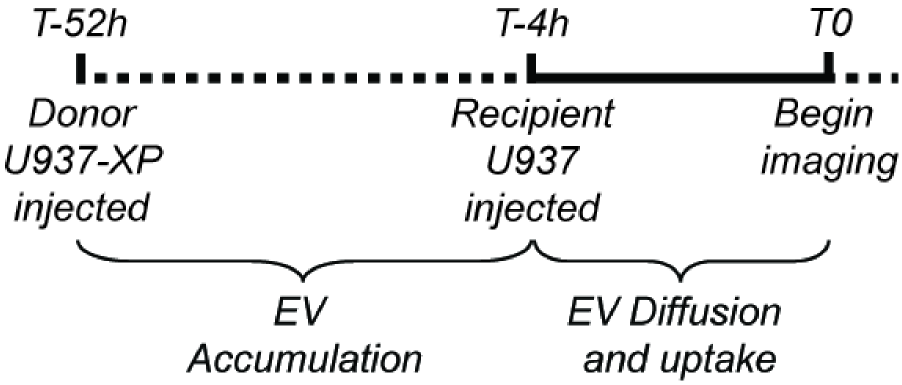

Figure S5. Timeline for cell injections in EV uptake experiment. Donor U937-XP cells were injected into the donor channel 52 hours prior to imaging to allow for EV accumulation in the donor and Matrigel channel. Recipient U937 cells were injected into the recipient channel 4 hours prior to imaging to allow time for EV diffusion into the recipient channel and uptake into recipient cells.

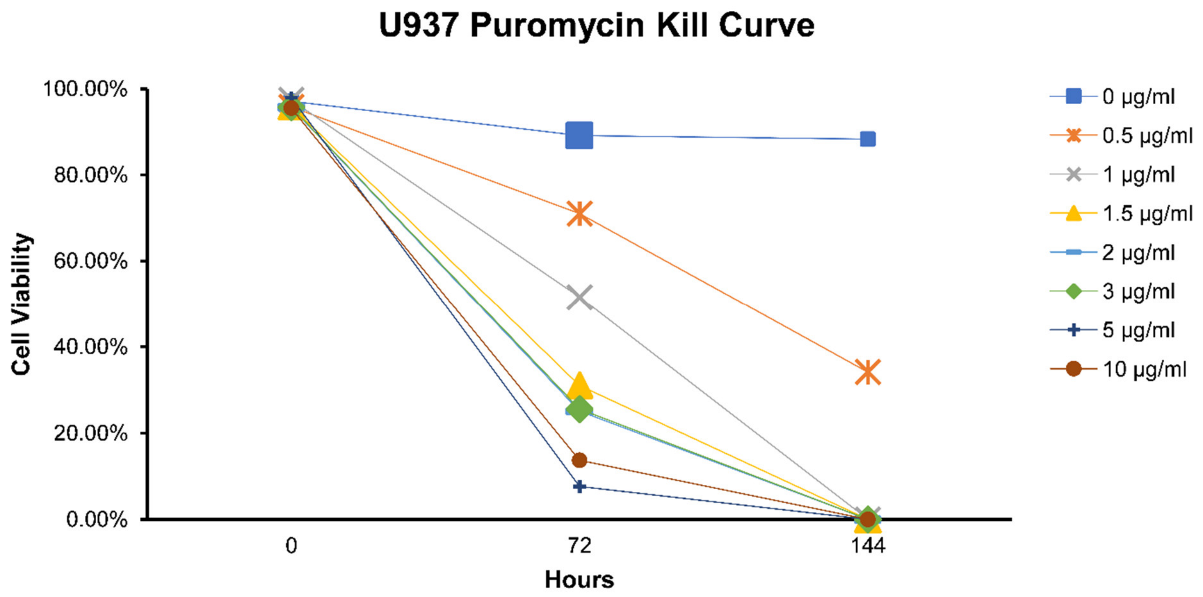

Figure S6. Puromycin kill curve for U937 cells. To determine the optimal concentration of puromycin to be used for antibiotic selection, multiple concentrations of puromycin were tested. For each concentration, cell viability was measured at times 0 hours, 72 hours, and 144 hours.

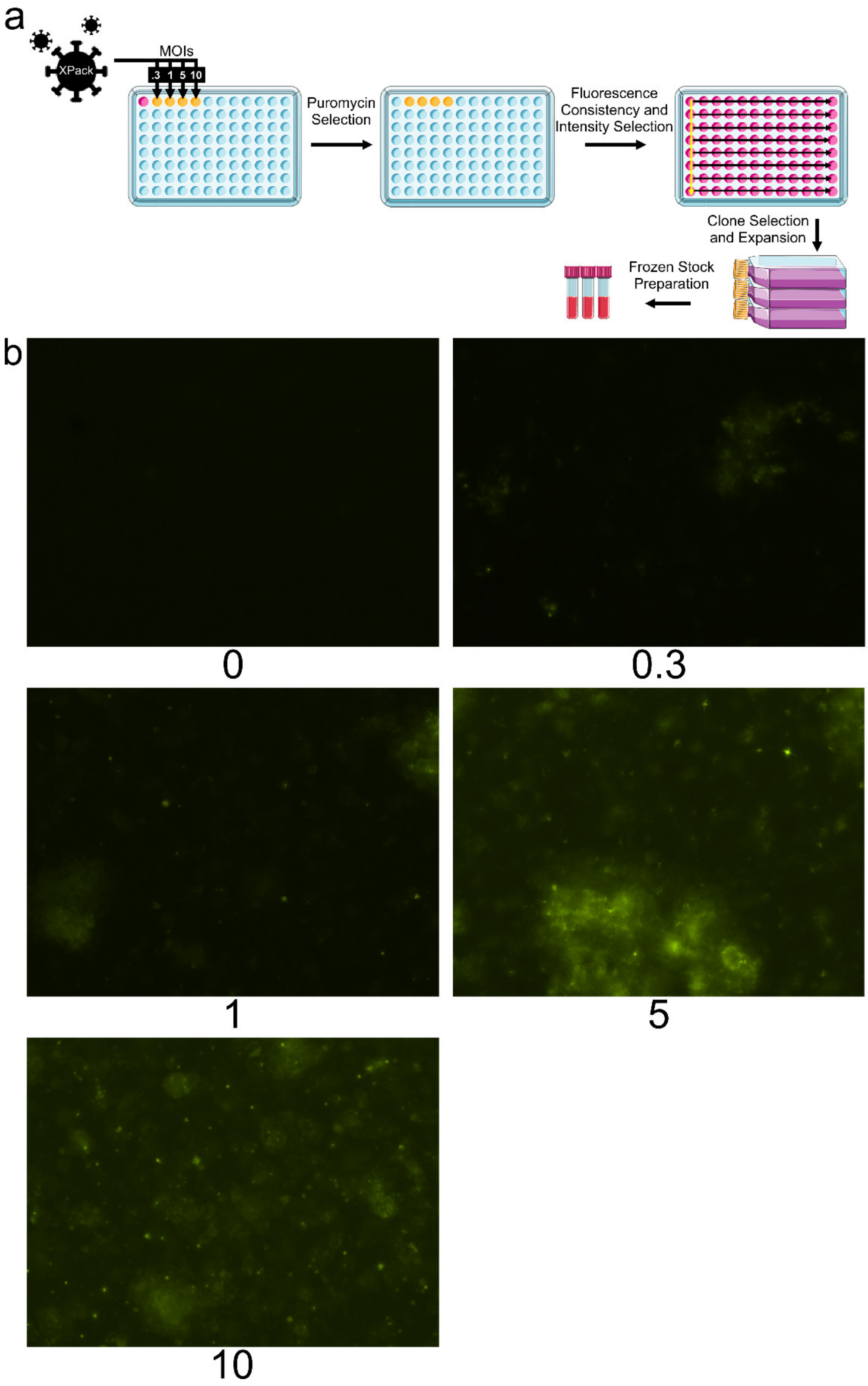

Figure S7. U937-XP cell line generation. a) Flowthrough diagram of the protocol used for generation of U937-XP, and b) fluorescent images of cells transduced at different MOIs obtained using GFP emission filter (original magnification: 40×).

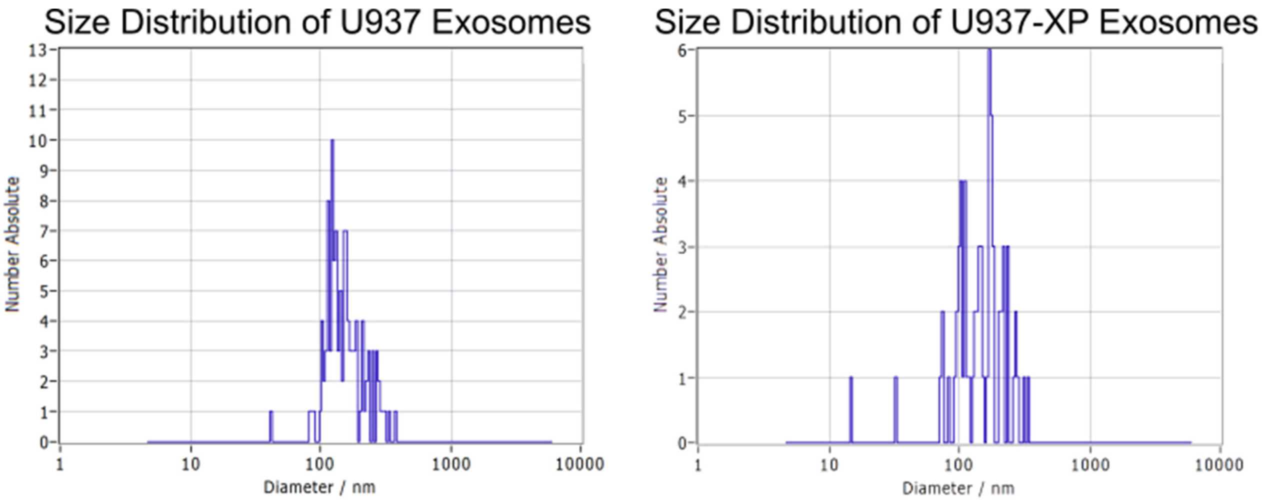

Figure S8. Size characterization of U937 and U937-XP exosomes. The right panel shows Zetaview nanoparticle tracking analysis (NTA) of exosomes purified from stable U937-XP cells; Original vesicle concentration: 4.98×10^8 particles/ml; Median diameter: 146.4 nm; Standard Deviation (S.D.): 64.6 nm. U937 exosomes were also analyzed by NTA and were found to be similar in size and concentration (Left); Original vesicle concentration: 5.2×10^8 particles/ml; Median diameter: 144.2 nm, S.D.: 59.3 nm.
